# Effect of washing, soaking, and cooking methods on perfluorinated compounds in mackerel (*Scomber japonicus*)

**DOI:** 10.1002/fsn3.1737

**Published:** 2020-07-08

**Authors:** Min‐Joo Kim, Jihyun Park, Li Luo, Juhyun Min, Jung Hoan Kim, Hee‐Deuk Yang, Younglim Kho, Gil Jin Kang, Myung‐Sub Chung, Sangah Shin, BoKyung Moon

**Affiliations:** ^1^ Department of Food and Nutrition Chung‐Ang University Anseong‐si, Gyeonggi‐do Korea; ^2^ Food Technology & Service Eulji University Seongnam‐si, Gyeonggi‐do Korea; ^3^ Department of Health Environment & Safety Eulji University Seongnam‐si, Gyeonggi‐do Korea; ^4^ Food Contaminants Division National Institute of Food and Drug Safety Evaluation Ministry of Food and Drug Safety Cheongju‐si Korea; ^5^ Department of Food Science and Technology Chung‐Ang University Anseong‐si, Gyeonggi‐do Korea

**Keywords:** cooking method, mackerel (*Scomber japonicus*), perfluorinated compounds, pretreatment, reduction

## Abstract

Perfluorinated compounds (PFCs) are environmental pollutants, and dietary intake is a major route of human exposure to them. We aimed to see the effects of washing, soaking, and cooking (grilling, braising, frying, and steaming) on the change of PFCs in mackerel fillets and PFCs before and after each treatment were analyzed using LC‐MS/MS. Washing resulted in a decrease in the PFC content of mackerel (average 74%) comparing to control. Among the 19 PFCs detected, perfluorobutanoic acid and perfluorotridecanoic acid (PFTrDA) were found to be abundant after washing. Soaking mackerel in sake reduced its PFC content by 51%, whereas soaking in rice‐washed solution reduced by 80% comparing to control. All the four cooking methods were effective in reducing the PFC content of mackerel. The degree by which the PFC content decreased varied with the cooking method: grilling (91%), steaming (75%), frying (58%), and braising (47%) comparing to uncooked sample. In addition, when mackerel was braised with potato, PFCs decreased more in fillet than the ones without potato. PFCs in potato increased after cooking with mackerel. The excessive consumption through the mackerel was 0.1997 ng/kg bw/day and 0.7987 ng/kg bw/day, respectively. These exposure levels were well below the tolerable daily intake values of both compounds (PFOS, 150 ng/kg bw/day; PFOA, 1,500 ng/kg bw/day). The results of this study indicated that employing appropriate pretreatment and cooking methods could be an effective way to reduce the dietary exposure to PFCs in mackerel.

## Graphical abstract

Soaking mackerel in rice‐washed water reduced its perfluorinated compound (PFC) content by 80%. PFCs in mackerel were reduced by cooking, and grilling was the most effect cooking method. It was suggested that employing appropriate pretreatment and cooking methods could be an effective way to reduce the dietary exposure to PFCs in mackerel.

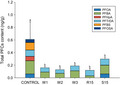

## 1 INTRODUCTION

Perfluorinated compounds (PFCs) have been found in air, water, soil, and house dust (Jogsten et al., 2009). They have been widely used in industrial products such as coating agents, varnishes (Prevedouros, Cousins, Buck, & Korzeniowski, 2006), and food packaging materials (Begley et al., 2005). Perfluorooctanoic acid (PFOA) and perfluorooctane sulfonate (PFOS) are the most stable and detectable PFCs (Lin, Panchangam, & Lo, 2009). The half‐life of PFOA in human body is reported to be about 4.37 years (Renner, 2003) and chronic exposure to it is reported to cause liver damage by fat metabolism (Vestergren, Cousins, Trudel, Wormuth, & Scheringer, 2008). Also, PFOS shows higher bioaccumulation than PFOA (Haug et al., 2010). European Food Safety Authority (EFSA) (2008) reported the tolerable daily intake (TDI) for PFOS and PFOA as 150 ng/kg bw/day and 1,500 ng/kg bw/day, respectively.

Haug et al. (2010) reported that food is an important source of exposure to PFCs in humans, and the main sources of chronic human exposure to PFCs are contaminated drinking water and food (Jogsten et al., 2009). Several studies have been conducted for determining the concentration of PFCs in food (Haug et al., 2010; Heo, Lee, Kim, & Oh, 2013; Kannan et al., 2002; Martin, Mabury, Solomon, & Muir, 2003) and their exposure levels for human body (Tittlemier, Pepper, & Edwards, 2006; Vestergren et al., 2008). Seafood has been reported to show a higher PFC content than other food items (Haug et al., 2010; Trudel et al., 2008), and Korea is one of the highest seafood consuming countries in the world (Choi et al., 2017). Though the dietary exposure to PFCs needs to be controlled (Jogsten et al., 2009), the study for the dietary exposure in Korean population is very limited. Therefore, it is important to monitor the amount of PFCs and analyze their changes during cooking in foods.

Mackerel (*Scomber japonicus*) has been the favorite and most popular fish in Korea with an average intake of 3.93 g per person per day (Korea Health Industry Development Institute, 2015; National Institute of Fisheries Science, 2010). It is consumed after various pretreatment (washing and soaking) and cooking method including sushi, grilling, braising, frying, and steaming. Also, mackerel is cooked with other ingredients such as potatoes, zucchini, and carrots according to preference (Kim, Baek, Kim, & Moon, 1998; Korean Food Promotion Institute, 2017). Potatoes contain high dietary fiber, which can be used as an absorbent to react with heavy metals (Kim, Shin, & Hwang, 1989; Lee & Lee, 1993). In Korea, rice‐washed solution and sake are commonly used as pretreatments for removing the unpleasant odor of fish. Rice‐washed solution has the advantage of being easily available because steamed rice is the main dish of Korea. Hong, Son, Kang, and Noh (2009) have reported that rice‐washed solution is a major source of domestic sewage. Therefore, the use of rice‐washed solution is an economical approach to remove the unpleasant odor of fish.

The results obtained from the studies carried out till date on the effect of cooking methods and pretreatment on the PFC content of fish are controversial. Del Gobbo et al. (2008) and Luo et al. (2019) have reported that the PFC concentration in fish, swimming crab, or fish cake decreased by cooking but the most effective cooking method to reduce its PFC content was different depending on the fish type. Jogsten et al. (2009) also reported that the PFC content in fish varied with their type and the method of cooking. On the other hand, Vassiliadou et al. (2015) reported that the PFC content in finfish and shellfish increased when they are fried or grilled. Therefore, further investigation is necessary to gain a clear understanding of the effect of cooking as well as washing and soaking methods on the PFC content of seafoods.

In this study, we aimed to investigate the effect of washing, soaking, and cooking methods on the PFC content in mackerel. For this purpose, different washing and soaking conditions as well as four cooking methods (grilling, braising, steaming, and frying) were investigated and the PFC concentrations before and after the washing, soaking, and cooking were analyzed using LC‐MS/MS.

## 2 MATERIALS AND METHODS

### 2.1 Chemicals

The method used here for the quantitative analysis of the PFCs present in mackerel could detect 19 PFCs: PFOS, perfluorodecane sulfonate (PFDS), perfluorohexane sulfonate (PFHxS), perfluorotetradecanoic acid (PFTeDA), perfluorotridecanoic acid (PFTrDA), perfluorododecanoic acid, perfluoroundecanoic acid, perfluorobutane sulfonate (PFBS), perfluorodecanoic acid (PFDA), perfluorononanoic acid (PFNA), perfluoroctanoic acid (PFOA), perfluorheptanoic acid (PFHpA), perfluorhexanoic acid (PFHxA), perfluorpentanoic acid (PFPeA), perfluorooctane sulfonamide (PFOSA), N‐ethyl‐perfluoro‐1‐octanesulfonamido acetic acid (N‐EtPFOSAA), N‐methylperfluoro‐1‐octanesulfonamido acetic acid (N‐MePFOSAA), sodium perfluoro‐1‐heptanesulfonate (L‐PFHpS), and perfluorobutanoic acid (PFBA) were purchased from Wellington Laboratories.

The internal standards for perfluoro‐n‐[1,2,‐^13^C_2_]hexanoic acid (^13^C_2_PFHxA), perfluoro‐n‐[1,2,3,4‐^13^C_4_]octanoicacid (^13^C_4_PFOA), perfluoro‐n‐[1,2,3,4,5‐^13^C_5_]nonanoic acid (^13^C_5_PFNA), perfluoro‐n‐[1,2‐^13^C_2_]decanoic acid (^13^C_2_PFDA), perfluoro‐n‐[1,2‐^13^C_2_]undecanoic acid (^13^C_2_PFUDA), perfluoro‐n‐[1,2‐^13^C_2_]dodecanoic acid (^13^C_2_PFDoA), perfluoro‐1‐hexane[^18^O_2_]sulfonic acid (^18^O_2_PFHxS), perfluoro‐1‐[1,2,3,4‐^13^C_4_]octanesulfonic acid (^13^C_4_PFOS), perfluoro‐n‐[1,2,3,4‐^13^C_4_]butanoic acid (^13^C_4_PFBA), N‐methyl‐d3‐perfluoro‐1‐octanesulfonamidoacetic acid (d3‐N‐EtFOSAA), N‐ethyl‐d5‐perfluoro‐1 octane sulfonamide acetic acid (d5‐N‐MeFOSAA), perfluoro‐n‐[1,2‐^13^C_2_]tetradecanoic acid (^13^C_2_PFTeDA), perfluoro‐n‐[1,2,3,4‐^13^C_4_]heptanoic acid (^13^C_4_PFHpA), perfluoro‐n‐[^13^C_5_]pentanoic acid (^13^C_5_PFPeA), and sodium perfluoro‐1‐[2,3,4‐^13^C_3_]butanesulfonate (^13^C_3_PFBS) were purchased from Wellington Laboratories. Water, hexane, MTBE (methyl‐tert‐butyl), acetonitrile, and methanol were obtained from Burdick & Jackson; protease, lipase, tetrabutylammonium hydrogen sulfate (TBAHS), sodium carbonate anhydrous (Na_2_CO_3_), sodium bicarbonate (NaHCO_3_), ammonium acetate, and formic acid were purchased from Sigma Aldrich.

### 2.2 Sampling

#### 2.2.1 Sampling of mackerel

Mackerel (*Scomber japonicus*, average 370 g, Norwegian) potatoes and rice (Icheon‐ssal, Gyeonggi‐do, Korea) were purchased from a retail market in Anseong (Korea). Fish organs, heads, and bones were removed using clean utensils and non‐PTFE (poly tetra fluoro ethylene) cutting boards following the method reported by Del Gobbo et al. (2008).

#### 2.2.2 Sample preparation and cooking

Mackerel were filleted and divided into three equal portions (upper, middle, and tail part) by mass. Each portion was analyzed to determine the PFC concentration in different parts of mackerel. Composite samples with three mackerel each were used to avoid bias due to interindividual variability.

In order to study the effect of washing and soaking on the PFC concentration in mackerel, ten mackerel were filleted and mixed into a composite sample, which was then divided into six equal portions by mass. One of these portions was left raw. The rice was mixed with tap water in a ratio of 1:2; then, rice grains were rubbed for 1 min in tap water. The milky white suspension was collected, and an equal amount of tap water was poured in and the rice‐washing was repeated two times more. The all rice‐washed solution was collected and mixed well for further using. Three portions were washed different times, and the remaining two portions were soaked for 15 min in rice‐washed solution (100 ml) and sake (200 ml), respectively. For the washing treatment, the fillets were washed (one, two, and three times) under running water for 20 s (at 75 ml/s).

To see the effect of cooking, the composite samples of 10 mackerel were divided into seven equal portions by mass (100 g each). One of these portions was left raw, two portions were grilled, two portions were braised, and the remaining two portions were fried and steamed, respectively. The recipes were devised from the preparatory experiments and suggestions from Korean rural development administration (Table 1). For cooking, stainless steel pan (SL, 26 cm diameter, 5 cm depth) was used for grilling, braising, and frying and stainless steel steamer pot (SLP, 26 cm diameter) was used for steaming.

**TABLE 1:** Recipes used to cook mackerel

	Ingredient	Cooking method	Ref
Grilling	Mackerel 1ea	Grill the mackerel for 6 min in the pan	Korea Rural Development Administration
	Edible oil 1Ts		http://terms.naver.com/entry.nhn?docId=1988385&cid=48164&categoryId=48202
Braising	Mackerel 1ea, water 1 cup, potato 100 g	Add potato, sauce, and water in a pot with mackerel and braise it for 25 min	Korea Rural Development Administration
	[sauce] soy sauce 2Ts, red pepper paste 2Ts, red pepper powder 1Ts, minced garlic 2Ts, minced ginger 1tsp, and sugar 1tsp,		http://www.nongsaro.go.kr/portal/ps/psr/psrc/useCkRyDtl.ps?menuId=PS03935&pageIndex=1&pageSize=10&pageUnit=10&cntntsNo=87115&ck_ry_ctg01=&ck_ry_ctg02=&schTrditfdNm
	Ground black pepper little		
Steaming	Mackerel 1ea Water 1 cup	Steam the mackerel for 10 min in a medium fire and 5 more min in a weak fire	Korea Rural Development Administration
			http://terms.naver.com/entry.nhn?docId=1627185&cid=48164&categoryId=48208
Frying	Mackerel 1ea	Fry the mackerel for 5 min at 160°C	Korea Rural Development Administration
	Edible oil 3 cups		http://terms.naver.com/entry.nhn?docId=1989983&cid=48164&categoryId=48206

**FIGURE 1:**
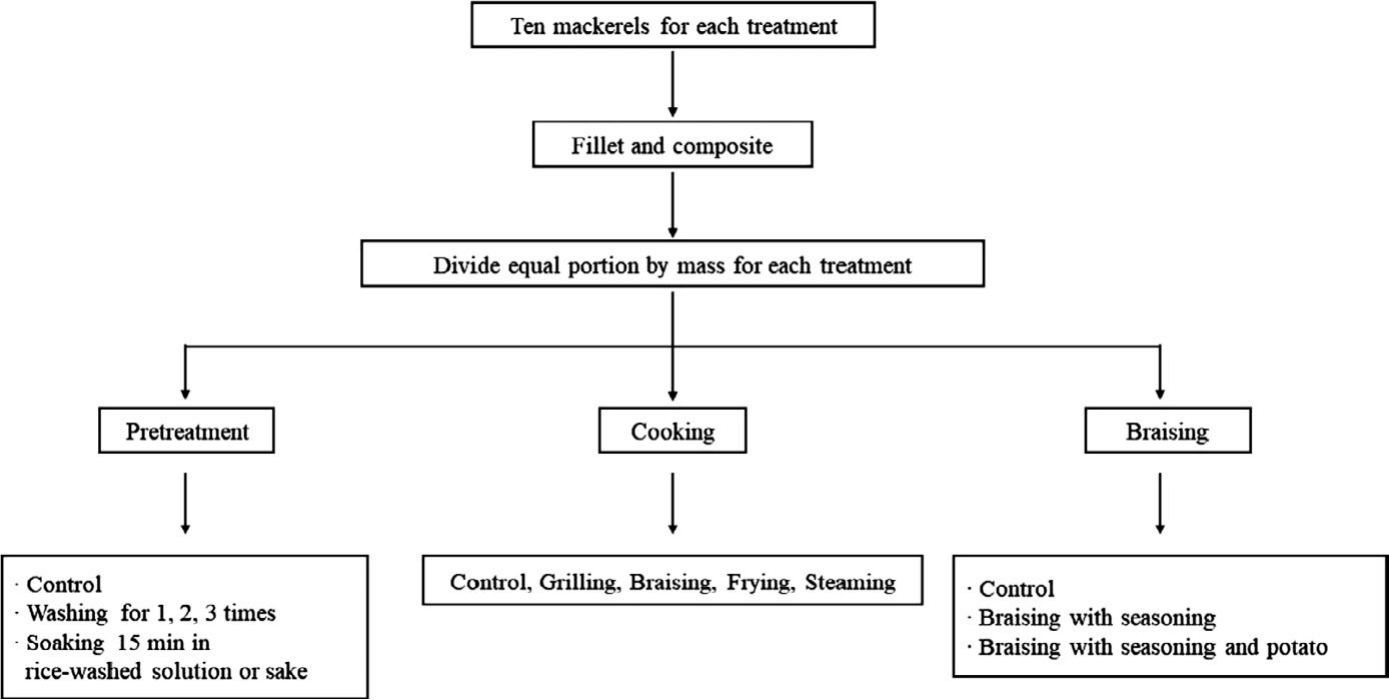
A schematic diagram of sample preparation for pretreatment and cooking of mackerel

To observe the transfer of PFCs from mackerel to ingredients during cooking, the composite samples were divided into three equal portions by mass. One of these portions was left raw, second portion was braised with only seasoning, and the remaining portion was braised with seasoning and potato. Potato which was selected as supplementary ingredient was sliced of 0.5 cm in thickness and braised in an amount of 100 g with mackerel fillet. Sampling procedure for pretreatment and cooking is shown in Figure 1. After cooking and pretreatments, all the samples were stored at −20°C for the analysis of PFCs.

### 2.3 Analytical method

The samples were extracted and analyzed for PFCs using the enzyme + hexane ion‐paring method reported by Bang et al. (2018). The samples were mixed with the same amount of distilled water, and the resulting mixtures were homogenized using a food processor (Tefal, BL1401 KR, Rumilly, France). After the addition of an internal standard solution mixture (20 μl), the homogenized samples (1 g) were mixed with protease (350 μl) and lipase (350 μl) in a polypropylene tube. The samples were hydrolyzed for 16 hr at 37°C in an incubator (Ilsin, Shaking incubator SH‐803R, Korea). Five millilitre of hexane was added, and sample was rotated for 15 min using a rotator (AG, FINEPCR, Rotator, Gyeonggi‐do, Korea). The samples were then centrifuged at 3,960 × *g* for 5 min (Gemmy Industrial Corp., PLC‐05, Taipei, Taiwan), and the supernatant was removed using a pipette (repeated two times). After the addition of 0.5 M TBAHS (1 ml), 0.25 M Na_2_CO_3_ (2 ml), and 0.25 M NaHCO_3_ (2 ml), the remnant was extracted using a sonicator (Bransonic, 5510R‐DTH) for 10 min. After 5 ml of MTBE was added, it was reextracted using rotator for 30 min and centrifuged at 11,001 × *g* for 5 min. The supernatant was collected in a new polypropylene tube and concentrated for 1 hr using a rotary evaporator (EYELA, CVE‐3100, UT‐1000, Tokyo, Japan) at 40°C. The concentrates were reconstituted with acetonitrile (200 μl) and injected into the LC‐MS/MS for analysis.

### 2.4 Instrumental analysis

An LC‐MS/MS system consisting of an Agilent 1100 LC series (Agilent Technologies) equipped with an Imtackt CD‐C C18 column (2.0 mm × 150 mm, 3.0 µm particle diameter; Imtakt, Kyoto, Japan), API 4000 spectrometer (Applied Biosystems), and electrospray ionization operating in negative mode was used. Analysis was conducted following the method by Luo et al. (2019). The mobile phase consists of 5 mM ammonium acetate with 0.02% formic acid in water (A) and methanol (B). The injection volume was 3 µl, and the flow rate was 200 µl/min. The gradient was linear from 30% to 100% solvent B in 5 min. For the next 8 min (until 13 min), the mobile phase consisted of 100% solvent B. The mobile phase composition was then varied from 100% to 30% solvent B over the period of 13–13.1 min. It was then kept constant at 30% solvent B until 25 min. The other experimental conditions used are as follows: an ion source gas 1, 40 psi; an ion source gas 2, 60 psi; an ion spray voltage of −4,500 V; and a gas temperature of 400°C. The analytical method for 19 PFCs using LC‐MS/MS was validated in terms of limits of detection (LOD) and quantification (LOQ), linearity, accuracy, and precision following the method by Luo et al. (2019).

### 2.5 Exposure assessment of PFCs

Dietary intake data were obtained from the Korea National Health and Nutrition Examination Survey (KNHANES 2015–2017), which were cross‐sectional and nationally representative surveys performed by Korea Centres for Disease Control and Prevention. The mean and the maximum daily consumption of PFOA and PFOS through mackerel were estimated base on the mean and the maximum PFOA and PFOS in each sample and mackerel intake data of 16,459 adults aged above 19 years. Dietary exposure to PFOA and PFOS in mackerel calculated using the following equation:

Mean/maximum daily intake=mean/maximum value of PFOA and PFOS concentration×daily consumption/body weight

For risk characterization, we used the TDI proposed by the EFSA (2008) as reference dose (1,000 ng/kg bw for PFOA and 150 ng/kg bw for PFOS).

### 2.6 Statistical analysis

The results were expressed as mean ± standard deviations (SDs). One‐way analysis of variance (ANOVA) is used each experimental set, and significance was defined at *p* < .05 by Duncan's multiple range test using SAS version 8.0 for Windows (SAS Inst).

## 3 RESULTS AND DISCUSSION

### 3.1 PFC accumulation in different parts of mackerel

**FIGURE 2:**
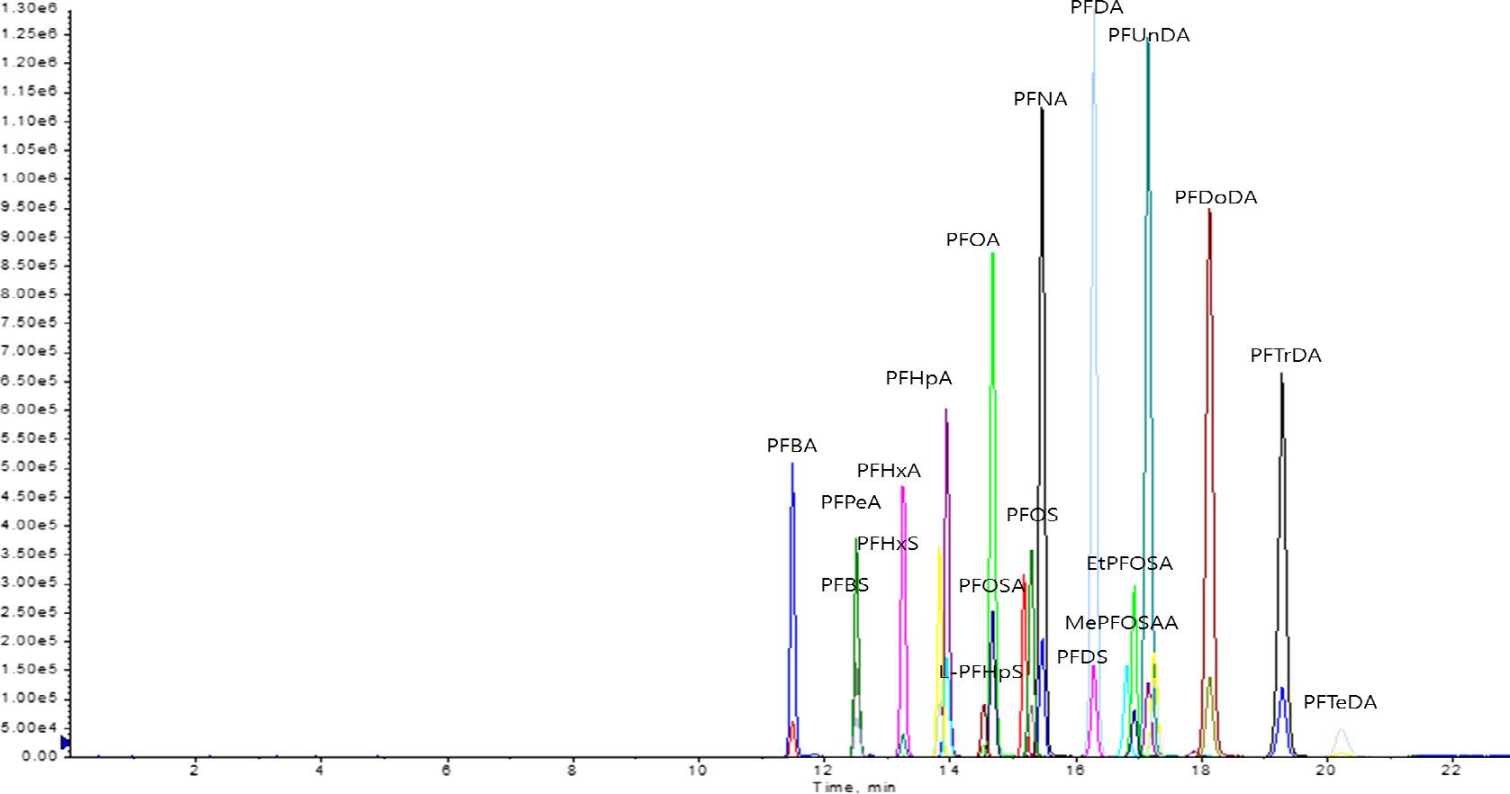
LC/MS/MS chromatograms of perfluorinated compounds in standard solution. PFBA, perfluorobutanoic acid; PFOA, perfluorooctanoic acid; PFUnDA, perfluoroundecanoic acid; PFPeA, perfluorpentanoic acid; PFBS, perfluorobutane sulfonate; PFHxA, perfluorhexanoic acid; PFHxS, perfluorohexane sulfonate; L‐PFHpS, sodium perfluoro‐1 heptanesulfonate; PFOS, perfluorooctane sulfonate; PFOSA, perfluorooctane sulfonamide; PFNA, perfluorononanoic acid; PFDA, perfluorodecanoic acid; MePFOSAA, methylperfluoro‐1 octanesulfonamido acetic acid; PFDS, perfluorodecane sulfonate; EtPFOSAA, ethyl‐perfluoro‐1 octanesulfonamido acetic acid; PFDoDA, perfluorododecanoic acid; PFTrDA, perfluorotridecanoic acid; PFTeDA, perfluorotetradecanoic acid; and PFHpA, perfluorheptanoic acid

**FIGURE 3:**
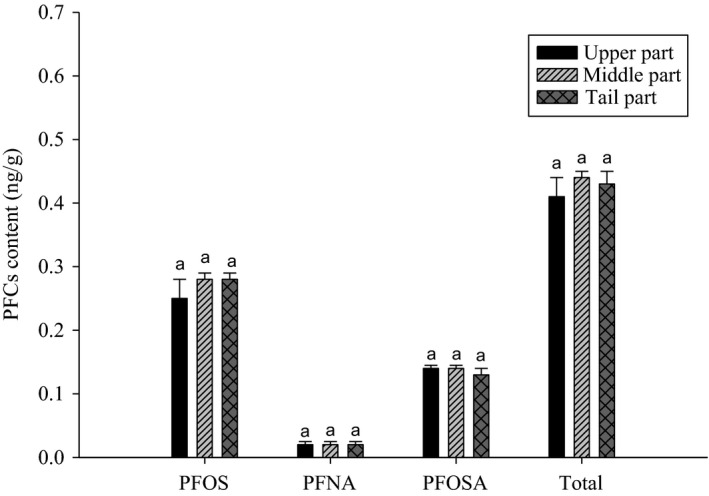
PFC contents (ng/g) in different parts of mackerel. Same letter means no significant difference at *p* < .05 by Duncan's multiple range test. PFOS, perfluorooctane sulfonate; PFNA, perfluorononanoic acid; and PFOSA, perfluorooctane sulfonamide

We validated analytical method for 19 PFCs and as reported in our previous research (Luo et al., 2019), for all 19 PFCs, linear regression equations showed good correlation coefficients (*R* ^2^ > .9976). LOQ and LOD ranged from 0.08 to 0.27 and 0.02 to 0.09 ng/g, respectively. The RSD values of interday and intraday variation were between 1.56% and 17.66%, and between 2.63% and 13.33%, respectively. For all 19 PFCs, the intraday accuracy ranged from 78.41% to 117.02% and the interday accuracy ranged from 78.61% to 118.30%. The concentration of PFCs in different parts of mackerel fillets was analyzed. LC/MS/MS chromatograms of PFCs in standard solution are shown in Figure 2. The results showed that the PFC levels in different parts of the mackerel samples were almost the same (Figure 3). Del Gobbo et al. (2008) reported that in fish, PFCs tend to accumulate more in liver than in muscle tissue. In addition, the PFC content in the liver and gut of fish is higher than that in its muscle tissue (Heo et al., 2013). Therefore, it is believed that the dietary exposure to PFCs can be reduced by avoiding the consumption of fish liver and gut. Furthermore, the level of PFC accumulation in different parts of the muscle tissue of fish is almost the same.

### 3.2 Effect of washing and soaking on PFC concentrations

**FIGURE 4:**
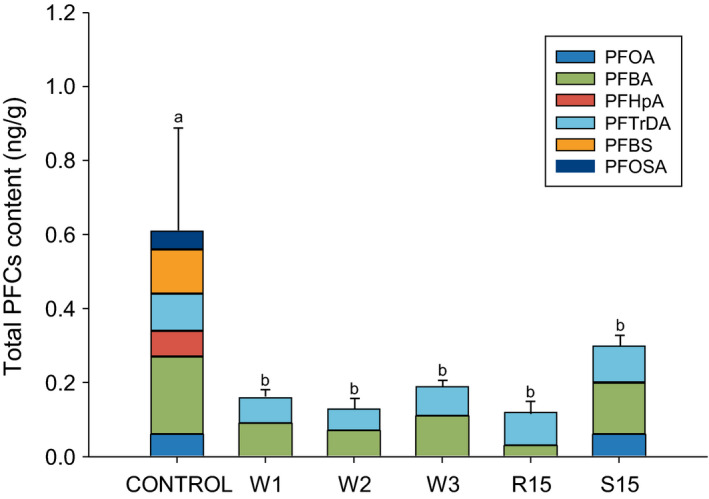
Efficacy of pretreatment on reducing the total PFC concentrations (ng/g) in mackerel. ^a,b^:Different letters indicate significant differences at *p* < .05 by Duncan's multiple range test. W1; washing once. W2; washing for two times. W3; washing for three times. S15; soaking in sake for 15 min. R15; soaking in rice‐washed solution for 15 min. PFBA, perfluorobutanoic acid; PFOA, perfluorooctanoic acid; PFHpA, perfluorheptanoic acid; PFTrDA, perfluorotridecanoic acid; PFBS, perfluorobutane sulfonate; and PFOSA, perfluorooctane sulfonamide

The effect of washing and soaking on the PFC concentration of raw mackerel was investigated, and the results are given in Figure 4. Washing is widely used to reduce the concentration of heavy metals or contaminants in food. Lee, Choi, and Park (2003) reported that around 20%–38% of heavy metals in oriental medical materials can be removed by simply washing them with water. In addition, Satpathy, Tyagi, and Gupta (2011) have reported that pesticide residues in vegetables can be reduced by washing (in various solutions). In this study, an average reduction of 74% was observed in the PFC concentration in mackerel after washing. A reduction of 74% was observed when the washing was carried out once (W1). When the washing was carried out twice, a reduction of 79% was observed (W2) and a reduction of 67% was observed when the washing was carried out three times (W3). Because of their high solubility in water (Prevedouros et al., 2006), the concentration of PFCs decreased after washing (Figure 4). This concentration reduction was not significantly affected by the washing frequency. In raw material, PFOA which has been reported to cause liver damage was not detected in any of the washed samples. On the other hand, no significant reduction was observed in the concentration of PFBA and PFTrDA after washing.

In Korea, the traditional method to remove the unpleasant odor of fish is to soak it in sake or rice‐washed solution (Hong et al., 2009; Woo, Choi, & Jeong, 2006). Soaking fish in liquid reduced PFC concentrations; soaking in sake reduced the PFC concentration by 51% and the PFC concentration reduced by 80% when rice‐washed solution was used for soaking. Among the pretreatment methods, soaking mackerel in rice‐washed solution was found to be the most effective approach to reduce their PFC concentration. The mackerel samples soaked in rice‐water solution for 15 min showed only PFBA and PFTrDA. In Korea, rice‐washed solution is commonly used for removing the unpleasant odor of fish. Therefore, soaking fish in rice‐washed solution is an effective approach to reduce its odor as well as PFC concentration.

### 3.3 Effect of cooking

In this study, the effect of cooking methods on the PFC concentration in fish was investigated. The PFC concentrations of mackerel after cooking are shown in Table 2. The total PFC content in raw mackerel (control, Norwegian) was 0.57 ± 0.14 ng/g, which is lower than that reported by Heo et al. (2013) for Korean mackerel (2.04 ± 0.57 ng/g). Seawater is exposed to PFCs either by direct contamination or by degradation of precursors in water (Herzke, Nygård, Berger, Huber, & Røv, 2009), and hence is considered a source of PFCs. Therefore, the PFC content in fish is affected by marine environment. Haug et al. (2010) also reported that species, age, and size of fish could cause large variations of the PFC concentrations in fish.

**TABLE 2:** Effect of cooking methods on the concentration of PFCs (ng/g) in mackerel

Compounds	Control	Grilled	Braised	Steamed	Fried
PFOA	0.07 ± 0.01^a^	ND	0.11 ± 0.00^a^	ND	0.09 ± 0.06^a^
PFOS	ND	ND	ND	ND	ND
PFBA	0.16 ± 0.01^a^	ND	0.13 ± 0.04^a^	0.14 ± 0.01^a^	0.09 ± 0.00^a^
PFPeA	ND	ND	ND	ND	ND
PFHxA	ND	ND	ND	ND	ND
PFHpA	0.07 ± 0.01^a^	ND	ND	ND	ND
PFNA	ND	ND	ND	ND	ND
PFDA	ND	ND	ND	ND	ND
PFUnDA	ND	ND	ND	ND	ND
PFDoDA	0.02 ± 0.01	ND	ND	ND	ND
PFTrDA	0.08 ± 0.01^a^	0.05 ± 0.00^b^	0.06 ± 0.02^b^	ND	0.06 ± 0.00^b^
PFTeDA	ND	ND	ND	ND	ND
PFBS	0.19 ± 0.01^a^	ND	ND	ND	ND
PFHxS	ND	ND	ND	ND	ND
L‐PFHpS	ND	ND	ND	ND	ND
PFDS	ND	ND	ND	ND	ND
PFOSA	ND	ND	ND	ND	ND
MePFOSAA	ND	ND	ND	ND	ND
EtPFOSAA	ND	ND	ND	ND	ND
Total	0.59 ± 0.06^a^	0.05 ± 0.00^d^	0.30 ± 0.06^b^	0.14 ± 0.01^cd^	0.24 ± 0.06^bc^

Note

Different letters in the same row indicate statistical difference (*p* < .05).

Abbreviations: and EtPFOSAA, ethyl‐perfluoro‐1 octanesulfonamido acetic acid;Control, raw mackerel; L‐PFHpS, sodium perfluoro‐1 heptanesulfonate; MePFOSAA, methylperfluoro‐1 octanesulfonamido acetic acid; ND, not detected; PFBA, perfluorobutanoic acid; PFBS, perfluorobutane sulfonate; PFDA, perfluorodecanoic acid; PFDoDA, perfluorododecanoic acid; PFDS, perfluorodecane sulfonate; PFHpA, perfluorheptanoic acid; PFHxA, perfluorhexanoic acid; PFHxS, perfluorohexane sulfonate; PFNA, perfluorononanoic acid; PFOA, perfluorooctanoic acid; PFOS, perfluorooctane sulfonate; PFOSA, perfluorooctane sulfonamide; PFPeA, perfluorpentanoic acid; PFTeDA, perfluorotetradecanoic acid; PFTrDA, perfluorotridecanoic acid; PFUnDA, perfluoroundecanoic acid.

The PFC content of all the mackerel samples decreased after cooking. In raw mackerel, PFBS were the most abundant and the concentration was 0.19 ng/g. PFOS was not detected in any of the samples, and PFOA was not detected in grilled and steamed samples. A reduction of 91% was observed in total PFC content when the mackerel samples were grilled. Braising reduced the PFC content by 47%, and reduction of 75% and 58% was observed after steaming and frying, respectively. Therefore, it is clear that all the cooking methods were helpful in reducing the PFC content in mackerel and that grilling was the most effective method. These results are in agreement with those reported by Del Gobbo et al. (2008). Cooking is widely used to eliminate toxins (Guzmán‐Guillén, Prieto, Moreno, Soria, & Cameán, 2011), heavy metals (Hwang, 2013), or pesticides (Jegal, Han, & Kim, 2000) from food. Wu et al. (2018) and Luo et al. (2019) reported that a cooking in high‐temperature heating with solvent (water or edible oil) would be effective at reducing PFCs with high polarity in food and this was in accordance with our results.

Potatoes are commonly used as supplementary ingredients to make braised mackerel (Kim et al., 1998). When braised with seasoning only, PFC content of mackerel fillet decreased by 41% comparing to raw fillet (Table 3). On the other hand, when it was cooked with potatoes, total PFC content of mackerel decreased by 79%. The addition of potatoes increased the reduction of PFCs compared to cooking with seasonings only. Also, in cooked potatoes, the contents of PFCs increased by 122%, comparing to uncooked potatoes (Table 3). Researches are being carried out to reduce heavy metals exposed to the natural environment by using agricultural products such as chestnut shell (Shin, Cha, Seo, & Kim, 1999), food‐fruit and oriental herb's (Kim, Oh, & Baek, 1999), and allium roots (Kim et al., 1998). Fiber, vitamins, proteins, phytin, and phenolic compounds are known as plant components that can react with heavy metals (Kim et al., 1998). Potatoes contain high dietary fiber, pectin, and phenolic compounds (Kim et al., 1989; Lee & Lee, 1993). Luo et al. (2019) reported similar result that PFCs in swimming crab decreased more when it was cooked with Korean radish which contains high fiber. It was considered that PFCs decreased when mackerel was cooked with potatoes because they were also adsorbed by dietary fibers in potatoes. Therefore, the results obtained in this study suggested that cooking was considered as an effective method to reduce the dietary intake of PFCs from mackerel.

**TABLE 3:** Change of PFC concentrations in braised mackerel by the addition of potato

Compound	Mackerel fillet			Potato	
	Uncooked	Cooked with Seasoning	Cooked with Seasoning + Potato	Uncooked	Cooked with Seasoning + Mackerel
PFOA	ND	ND	ND	ND	ND
PFOS	ND	0.11 ± 0.20^a^	ND	ND	ND
PFBA	0.20 ± 0.01^a^	0.12 ± 0.01^b^	0.08 ± 0.02^c^	0.09 ± 0.02^a^	0.12 ± 0.04^a^
PFPeA	ND	ND	ND	ND	ND
PFHxA	ND	ND	ND	ND	ND
PFHpA	ND	ND	ND	ND	ND
PFNA	ND	ND	ND	ND	ND
PFDA	ND	ND	ND	ND	ND
PFUnDA	0.11 ± 0.00^a^	ND	ND	ND	ND
PFDoDA	0.02 ± 0.01^a^	ND	ND	ND	0.08 ± 0.00^b^
PFTrDA	0.08 ± 0.01^a^	ND	ND	ND	ND
PFTeDA	ND	ND	ND	ND	ND
PFBS	ND	ND	ND	ND	ND
PFHxS	ND	ND	ND	ND	ND
L‐PFHpS	ND	ND	ND	ND	ND
PFDS	ND	ND	ND	ND	ND
PFOSA	ND	ND	ND	ND	ND
MePFOSAA	ND	ND	ND	ND	ND
EtPFOSAA	ND	ND	ND	ND	ND
Total	0.41 ± 0.03^a^	0.23 ± 0.21^ab^	0.08 ± 0.02^b^	0.09 ± 0.02^b^	0.20 ± 0.04^a^

Note

Different letters in the same row indicate statistical difference (*p* < .05).

Abbreviations: and MePFOSAA, methylperfluoro‐1 octanesulfonamido acetic acid; L‐PFHpS, sodium perfluoro‐1 heptanesulfonate; ND, not detected. PFOA, perfluorooctanoic acid; PFBA, perfluorobutanoic acid; PFBS, perfluorobutane sulfonate; PFDA, perfluorodecanoic acid; PFDoDA, perfluorododecanoic acid; PFDS, perfluorodecane sulfonate; PFHpA, perfluorheptanoic acid; PFHxA, perfluorhexanoic acid; PFHxS, perfluorohexane sulfonate; PFNA, perfluorononanoic acid; PFOS, perfluorooctane sulfonate; PFOSA, perfluorooctane sulfonamide; PFPeA, perfluorpentanoic acid; PFTeDA, perfluorotetradecanoic acid; PFTrDA, perfluorotridecanoic acid; PFUnDA, perfluoroundecanoic acid.

### 3.4 Dietary PFOA and PFOS exposure assessment

The mean daily intake of mackerel was 0.9 g/day for whole Korean adults aged above 19 years old and 42.5 g/day for consumer only. The 99th percentile consumption of mackerel was 36.8 g/day for whole Korean adults aged above 19 years old and 185.0 g/day for consumer only. The mean exposure to PFOs using mean intake of 42.5 g/day (consumer only) with mean concentration of 0.03 μg/kg for PFOA and 0.03 μg/kg for PFOS was 0.0197 × 10^–3^ μg/kg bw/day and 0.0197 ng/kg bw/day, respectively. The excessive exposure to PFOs using large intake of 185.0 g/day (P99 for consumer only) with maximum concentration of 0.07 μg/kg for PFOA and 0.28 μg/kg for PFOS was 0.1997 ng/kg bw/day and 0.7987 ng/kg bw/day, respectively (Table 4). It was lower than the recommended TDI (1,000 ng/kg b.w. for PFOA and 150 ng/kg bw for PFOS) proposed by the EFSA. Chub mackerel is the most popular fish in Korea and Japan, and mackerel is consumed more than 800 million tons in Europe (Bae & Lim, 2012; FAO, 2018). Also, Indian mackerel (*Rastreliiger Kanagurta*) is the most important fishing resources in South‐East Asian countries (Wu, Pu, & Sun,  2019). Therefore, it is important to estimate the dietary PFOA and PFOS exposure by mackerel.

**TABLE 4:** Dietary exposure to PFOA and PFOS according to the consumption of mackerel and concentration data

Compounds	Concentration data		Mackerel consumption data		Body weight (kg)	Exposure (ng/kg bw/day)	Reference dose: TDI (ng/kg bw)
	Type	Values (μg/kg)	Type	Values (g/day)			
PFOA	Mean	0.03	Mean consumption (whole adults)	0.9	64.9	0.0004	1,500
	Max.	0.07	Consumption P99 (whole adults)	36.8	64.9	0.0397	
	Mean	0.03	Mean consumption (consumers only)	42.5	64.8	0.0197	
	Max.	0.07	Consumption P99 (consumers only)	185.0	64.8	0.1997	
PFOS	Mean	0.03	Mean consumption (whole adults)	0.9	64.9	0.0004	150
	Max.	0.28	Consumption P99 (whole adults)	36.8	64.9	0.1588	
	Mean	0.03	Mean consumption (consumers only)	42.5	64.8	0.0197	
	Max.	0.28	Consumption P99 (consumers only)	185.0	64.8	0.7987	

Abbreviations: PFOA, perfluorooctanoic acid; PFOS, perfluorooctane sulfonate; TDI, tolerable daily intake.

Additionally, we evaluated the excessive exposure to PFOs using data of mackerel consumption P99 and maximum concentration across different age groups. The exposure to PFOA across different age groups was estimate to be a minimum of 0.1243 ng/kg bw/day aged 30–39 years and a maximum of 0.2532 ng/kg bw/day aged 50–59 years. The exposure to PFOS was calculated to be 0.4970–1.0129 ng/kg bw/day. It was shown no significant differences among age groups (Table 5). Consequently, exposure assessment to PFOs through the mackerel consumption according to each age group estimated to be an intake of PFOs lower than the TDI.

**TABLE 5:** Dietary exposure to PFOA and PFOS according to the consumption of mackerel in different age groups for consumer only

Compounds	Age (years)	Body weight (kg)	Consumption P99 (g/day)	Max. concentration (μg/kg)	Exposure (ng/kg bw/day)	Reference dose: TDI (ng/kg bw)
PFOA	19–29	72.63	150.64	0.07	0.1452	1,500
	30–39	67.45	119.73	0.07	0.1243	
	40–49	66.77	146.80	0.07	0.1539	
	50–59	63.56	229.93	0.07	0.2532	
	60–69	62.18	172.36	0.07	0.1941	
	70+	59.12	177.92	0.07	0.2107	
PFOS	19–29	72.63	150.64	0.28	0.5807	150
	30–39	67.45	119.73	0.28	0.4970	
	40–49	66.77	146.80	0.28	0.6156	
	50–59	63.56	229.93	0.28	1.0129	
	60–69	62.18	172.36	0.28	0.7762	
	70+	59.12	177.92	0.28	0.8426	

Abbreviations: PFOA, perfluorooctanoic acid; PFOS, perfluorooctane sulfonate; TDI, tolerable daily intake.

## 4 CONCLUSIONS

In this study, the effect of pretreatment (washing and soaking) and cooking (grilling, braising, frying, and steaming) methods on the PFC content of mackerel was investigated. The PFC contents of the raw, pretreated, and cooked mackerel samples were determined. An average reduction of 74% was observed in the PFC content of mackerel when it was washed with water (one, two, and three times). Soaking in sake and rice‐washed solution reduced the PFC content of mackerel by 51% and 80% comparing to control, respectively. All the four cooking methods were effective in reducing the PFC content of mackerel. The grilled, steamed, fried, and braised samples showed a PFC content reduction of 91%, 75%, 58%, and 47% comparing to uncooked sample, respectively. In addition, when mackerel was braised with potato, PFCs decreased more in mackerel fillet and increased in potato. The estimated excessive dietary intake of 0.1997 ng/kg bw/day of PFOA and 0.7987 ng/kg bw/day of PFOS for Korean adult consumers was well below the TDI set by EFSA. Based on the results of this study, employing appropriate pretreatment and cooking methods was suggested to be an effective way to reduce the dietary exposure to PFCs in mackerel.

## 5 INFORMED CONSENT

None.

## CONFLICT OF INTEREST

All authors declare that they do not have any conflict or interest.

## ETHICAL STATEMENT

This study does not involve any human or animal testing.
